# Advanced data analysis and prediction model for student mental health risk assessment

**DOI:** 10.3389/fpsyg.2025.1682083

**Published:** 2025-11-11

**Authors:** Xiuyu Shi, Jin Pan, Daofu Yuan, Minye Li, Yafeng Pan

**Affiliations:** 1 Department of Psychology and Behavioral Sciences, Zhejiang University, Hangzhou, Zhejiang, China; 2 Zhejiang Institute of Communications, Hangzhou, Zhejiang, China; 3 School of Psychology, Zhejiang Normal University, Jinhua, Zhejiang, China

**Keywords:** student mental health, multi-modal data fusion, graph convolutional networks, self-supervised learning, temporal modeling, early detection and intervention

## Abstract

With the increasing prevalence of mental health issues among students, early detection plays a crucial role in ensuring timely intervention. Existing methods struggle to capture the complex relationships among diverse data sources, such as behavioral, emotional, and physiological data, and fail to account for the temporal dynamics of mental health changes. This study addresses these challenges by proposing PsyGraph-SSL, a novel model that combines graph convolutional networks (GCN), temporal modeling, and self-supervised learning (SSL) to predict and analyze student mental health risks. The PsyGraph-SSL model integrates multi-modal data, including emotional, behavioral, and physiological signals, and learns temporal dependencies through time-series modeling. It employs GCN for processing social relationships and emotional interactions, while SSL is utilized to leverage unlabeled data and enhance feature learning. Temporal modeling further captures dynamic changes in students' mental health status, providing both short-term and long-term predictions. Experimental results on the WESAD and Student Well-Being Dataset show that PsyGraph-SSL outperforms traditional models, achieving higher accuracy, F1 score, AUC, and other key metrics. The model demonstrates strong performance in capturing emotional and behavioral fluctuations, making it highly effective for early detection and intervention. PsyGraph-SSL offers a comprehensive solution for student mental health monitoring, highlighting the importance of multi-modal data fusion and temporal analysis. The experimental results validate the model's potential for providing real-time, adaptive support. Future work will focus on expanding the dataset, improving generalization, and addressing challenges such as data imbalances and noise to further enhance the model's practical applicability.

## 1 Introduction

With the rapid development of society and increasing academic pressure, students' mental health issues are receiving increasing attention. Mental health not only affects students' academic performance but also profoundly impacts their physical and mental development (Chen, 2024; Chung and Teo, 2022), social interactions, and future careers. Therefore, how to effectively monitor and assess students' mental health risks has become a critical topic in the fields of education and psychology. However, traditional mental health assessment methods (Tutun et al., 2023), such as questionnaires and face-to-face interviews, suffer from subjectivity, difficulty in quantifying data, and long assessment cycles, making it difficult to comprehensively and timely reflect changes in students' mental states (Thieme et al., 2020; Whiting et al., 2021). These shortcomings make it difficult for many schools to promptly identify potential mental health issues, missing the optimal opportunity for intervention.

With the rapid development of information technology, deep learning technology has been widely applied in various fields and has provided new insights for mental health assessment (Córdova Olivera et al., 2023). In particular, the combination of multimodal data analysis and time series modeling techniques has made data-based mental health risk prediction possible (Jiang et al., 2020; Chen et al., 2020). However, most existing methods primarily focus on single-modal data, such as speech recognition, facial expression analysis, and social media text analysis, which have limitations in terms of dynamicity and personalized prediction (Bickman, 2020). These methods often fail to capture the full complexity of emotional and behavioral fluctuations, leading to insufficient predictive power (Kizilcec, 2024; Alqahtani et al., 2023). In contrast, this paper proposes a novel approach that integrates multimodal data (including emotional, behavioral, and physiological signals) and time-series modeling to more accurately predict mental health risks by accounting for dynamic changes over time.

Furthermore, data scarcity remains a significant challenge for current mental health prediction models (Visser and Law-van Wyk, 2021). While increasing amounts of mental health data are being collected and made publicly available, the lack of labeled data still limits the effectiveness of deep learning model training (Aafjes-van Doorn et al., 2021). In particular, individual differences and emotional fluctuations in student mental health analysis are highly personalized, and most existing models rely on manual annotation and are unable to fully utilize unlabeled data (Werner et al., 2021). Therefore, how to effectively extract and learn mental health features using deep learning algorithms with limited or no labeled data remains an urgent challenge.

To address these challenges, this paper proposes a hybrid model based on graph convolutional networks and self-supervised learning, PsyGraph-SSL, designed to improve the accuracy and timeliness of student mental health risk assessment within a framework of multimodal data fusion and time series prediction. Specifically, we will utilize graph convolutional networks (GCNs) to process social relationship data among students, capturing the spread and evolution of group mental health status. We will also introduce self-supervised learning (SSL) mechanisms to effectively learn features from unlabeled data, overcoming the scarcity of labeled data. Furthermore, based on time series modeling, we can capture dynamic changes in students' mental health in real time and predict both long-term and short-term risks. This model not only considers students' immediate mental health status but also provides personalized risk assessments, thereby providing more accurate and timely support for psychological intervention.

The innovations of this paper can be summarized as follows:

1. Combining a graph convolutional network (GCN) with a self-supervised learning (SSL) model, this approach leverages graph structures to capture mental health relationships among students and effectively processes unlabeled data through self-supervised learning.
2. A multimodal data fusion strategy comprehensively considers multiple data sources, including text, speech, and behavior, to improve the accuracy of mental health risk prediction.
3. Based on time series modeling, this approach captures the dynamic changes in students' mental health status and predicts future mental health risk trends.
4. This approach provides personalized mental health risk assessment and intervention support, overcoming the limitations of existing models in personalized analysis.

This paper is organized as follows: Section 2 reviews relevant research and literature, introducing the limitations of current student mental health assessment methods and the application of deep learning techniques in this field. Section 3 details the proposed PsyGraph-SSL model, including its overall framework, GCN module, SSL module, and implementation of time series modeling. Section 4 presents the experimental design and results analysis, focusing on the dataset used, experimental setup, evaluation metrics, and a discussion of the experimental results. Finally, Section 5 summarizes the research findings and proposes future research directions.

## 2 Related work

### 2.1 Research progress on traditional mental health assessment and intervention methods

The assessment and intervention of student mental health has always been a key topic in educational psychology and mental health research. Traditional mental health assessment methods typically rely on self-report questionnaires (Kolenik, 2022), interviews, and behavioral observations. While widely used, these methods also have significant limitations. For example, self-report questionnaires require students to provide their own emotional experiences and mental states, which is susceptible to subjective factors and can lead to biased results (Olawade et al., 2024; Howard and Khalifeh, 2020). Furthermore, interviews often rely on the experience and professional judgment of psychologists, which may have varying applicability to students from different cultural backgrounds and social environments.

With the continuous development of mental health assessment technology, behavioral observation has gradually become an effective assessment tool. By observing students' daily behavior, social interactions, and stress coping strategies, researchers can indirectly infer their mental health status (Eaton et al., 2023). Although this method can objectively reflect students' psychological performance, it is also limited by the observer's judgment criteria and the contextual variations during observation, lacking sufficient standardization and quantification (Jay et al., 2024; Liu et al., 2024). Therefore, while traditional assessment methods can assess students' mental health to a certain extent, their subjectivity and timeliness have always limited their effectiveness.

In terms of mental health intervention, traditional methods mainly focus on face-to-face counseling and group therapy. Face-to-face counseling, often provided by professional psychological counselors, aims to help students overcome their problems and relieve psychological stress through psychological counseling (Schleider et al., 2020). However, this type of intervention is often limited by time and space, and due to the limited number of intervention subjects, it is difficult to meet the needs of a large number of students. Group therapy provides psychological support through collective means, but for students with significant individual differences, this collective intervention may not be as effective as personalized intervention (Karney and Bradbury, 2020). In general, while traditional mental health intervention methods can help students alleviate psychological problems in the short term, their effectiveness is limited by the single intervention method and insufficient resources.

### 2.2 Early prediction and intervention of student mental health risks

Student mental health issues often exhibit gradual and hidden characteristics. Many problems do not manifest obvious symptoms in the early stages and only attract attention when symptoms intensify. Therefore, early prediction and timely intervention are key to mental health management (Adnan et al., 2021). With the advancement of psychological research, traditional mental health assessment methods are gradually being replaced by modern technologies, particularly data-driven prediction methods, which can effectively detect early signs of student mental health issues (Sheldon et al., 2021). The core of early prediction lies in the timely identification of potential mental health risks and the provision of personalized intervention strategies based on the predicted results, thereby effectively reducing the incidence of mental health issues.

Early prediction methods typically rely on information such as student behavioral data, emotional changes, and physiological signals. In recent years, many studies have begun to attempt to predict mental health risks by analyzing students' social network behavior, learning patterns, and emotional fluctuations. For example, social media text analysis is widely used to detect emotional changes (Wu et al., 2020). Fluctuations in academic performance, participation in extracurricular activities, and peer interactions are also considered early signs of student mental health. By analyzing these data, researchers can detect signs of mental health issues in the early stages of a student's development and implement preventive interventions. Compared to traditional questionnaires and face-to-face interviews, early predictions based on behavioral data are more real-time and comprehensive (Bettini et al., 2020), effectively capturing subtle changes in students' mental health.

In terms of early intervention, personalized intervention has become a key direction in modern mental health management. Unlike traditional group therapy and standardized psychological intervention programs, personalized intervention considers students' specific needs and individual differences, providing tailored solutions. Personalized intervention is based on accurate risk prediction to identify key factors associated with mental health issues and select the most appropriate intervention approach based on students' diverse backgrounds and mental states (Cuthbert, 2022). In recent years, methods based on data analysis and deep learning have been applied to personalized intervention design. By analyzing students' behavioral and emotional data, customized intervention strategies are provided for each student. This intervention approach not only addresses students' mental health issues promptly but also avoids excessive or inappropriate intervention.

### 2.3 Affective computing and mental health analysis

Affective computing, a key research area at the intersection of artificial intelligence and psychology, has been widely used in mental health analysis in recent years. Affective computing technology analyzes an individual's emotional state to help identify their mental health (Smith et al., 2021). Changes in emotional state are often closely related to mental health, particularly in areas such as mood swings, emotional disorders, and excessive stress. Abnormal emotional fluctuations are often precursors to mental health issues (Fei et al., 2020). Therefore, affective computing offers a new approach to student mental health assessment, enabling early identification of potential mental health issues.

Core technologies in affective computing include speech emotion analysis, facial expression recognition, and text emotion analysis. These technologies monitor students' emotional states in real time by analyzing their voice intonation, facial expressions, and the language content of social media and daily interactions. For example, speech emotion analysis can identify emotional changes in students during phone calls (De Melo et al., 2020), online interactions, or face-to-face conversations. Facial expression analysis uses image recognition to capture subtle fluctuations in facial expressions, which are often closely related to their emotional state. Furthermore, text emotion analysis can further identify potential mental health risks by analyzing the emotional tendencies in students' written language (Saganowski et al., 2022). Combining multiple emotional computing technologies can more comprehensively assess students' mental health status, especially timely capture the mental health risks behind their emotional changes.

## 3 Method

### 3.1 Overview of our network

**Figure 1:**
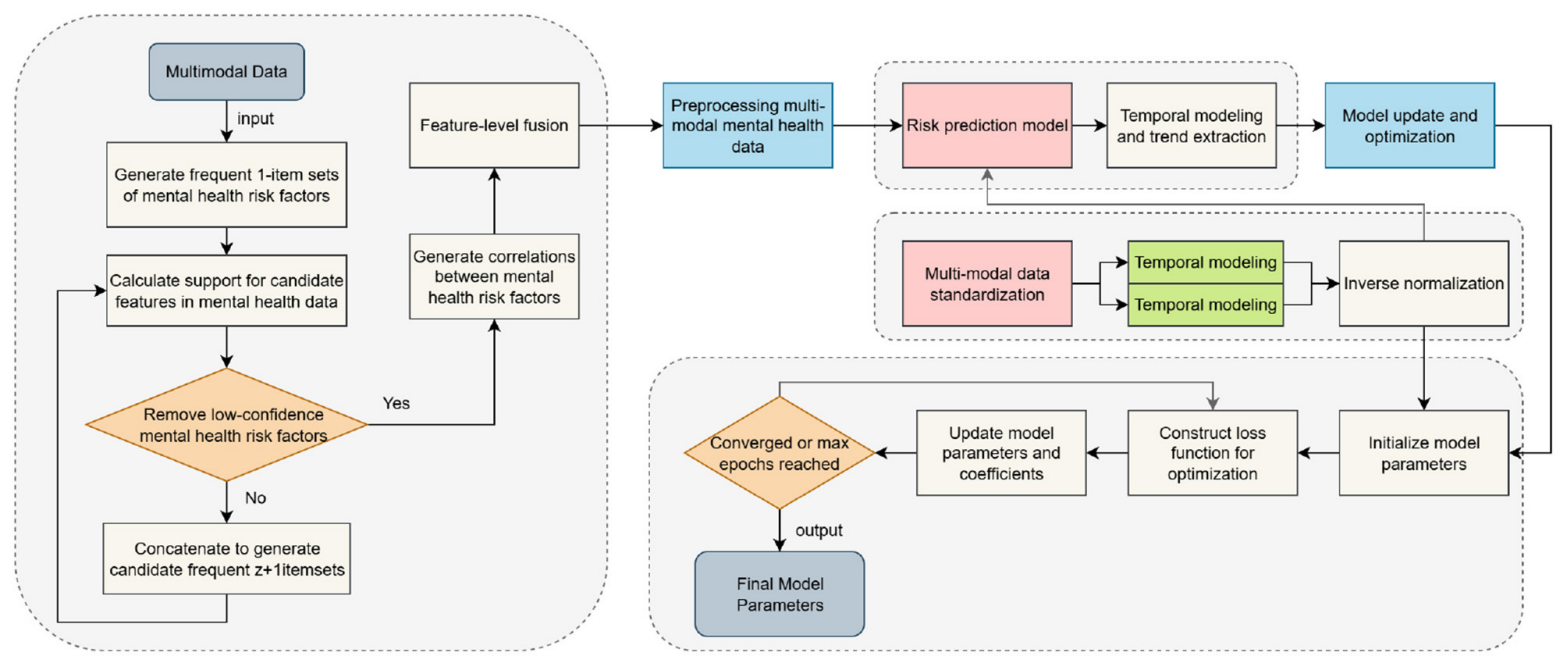
PsyGraph-SSL model architecture.

The model proposed in this paper, PsyGraph-SSL, combines two deep learning techniques: graph convolutional networks (GCNs) and self-supervised learning (SSL). It aims to accurately predict students' mental health risks through multimodal data fusion and time series modeling. The overall architecture of the model is shown in Figure 1. From input to output, various modules work together to analyze and assess students' mental health status.

The model input includes data from multiple sources, including student behavior, speech, facial expression, and sentiment analysis text data. First, time series data from students' daily behavior and social interactions are processed by the graph convolutional network (GCN) module. The GCN's task is to model the internal relationship network of the student group and capture the spread of mental health status and emotional interactions among students. For example, the interactions and emotional connections between students and their classmates have a profound impact on their mental health, and these relationships are effectively captured through a graph structure. The graph convolutional network transforms this social and emotional information into node features within the graph structure, providing important information for subsequent mental health prediction.

Meanwhile, speech and facial expression data are analyzed by the emotion computing module. Emotion recognition from voice data can help capture the dynamics of students' emotions, while facial expression data provides visual evidence of their emotional fluctuations. By analyzing this multimodal data, the model can provide a more comprehensive assessment of students' emotional states and mental health from multiple dimensions. The outputs of these emotion calculation modules are converted into features that can be used for model decision-making, providing further detailed information about students' emotional fluctuations.

Next, all features from different data sources enter the self-supervised learning (SSL) module. The SSL module uses unlabeled data to learn features and further explore potential patterns and information in the data. At this stage, the model automatically learns deep-level features of students' mental health through self-supervised tasks such as contrastive learning and generative tasks, without relying on manually annotated labels. This approach enables efficient training even on smaller labeled datasets, overcoming data scarcity.

The features after self-supervised learning are then passed to the time series modeling module. Using time series models such as the Transformer, the time series modeling module models the changing trends of students' mental health. By capturing long-term dependencies in students' mental health, this module can predict students' mental health risks in real time over a period of time. For example, students' mood swings and behavioral changes are often gradual. The time series modeling module can identify trends in these changes and predict their future mental health.

Finally, all features processed through multiple layers are passed to the model's output layer, which outputs the student's mental health risk score. Based on the student's multi-dimensional emotional and behavioral data, combined with their interactions within their social network, the model assesses their current mental health status and provides short- and long-term risk predictions. These results not only help educators identify potential mental health issues but also provide data support for subsequent personalized interventions.

Through this comprehensive approach, which integrates multimodal data processing, graph convolutional networks, affective computing, self-supervised learning, and time series modeling, our model provides a comprehensive and accurate student mental health risk assessment system. The synergy between these modules not only enables efficient data processing but also enables effective mental health predictions without relying on large amounts of labeled data, providing reliable technical support for personalized interventions and early warning systems.

### 3.2 Graph convolutional networks

**Figure 2:**
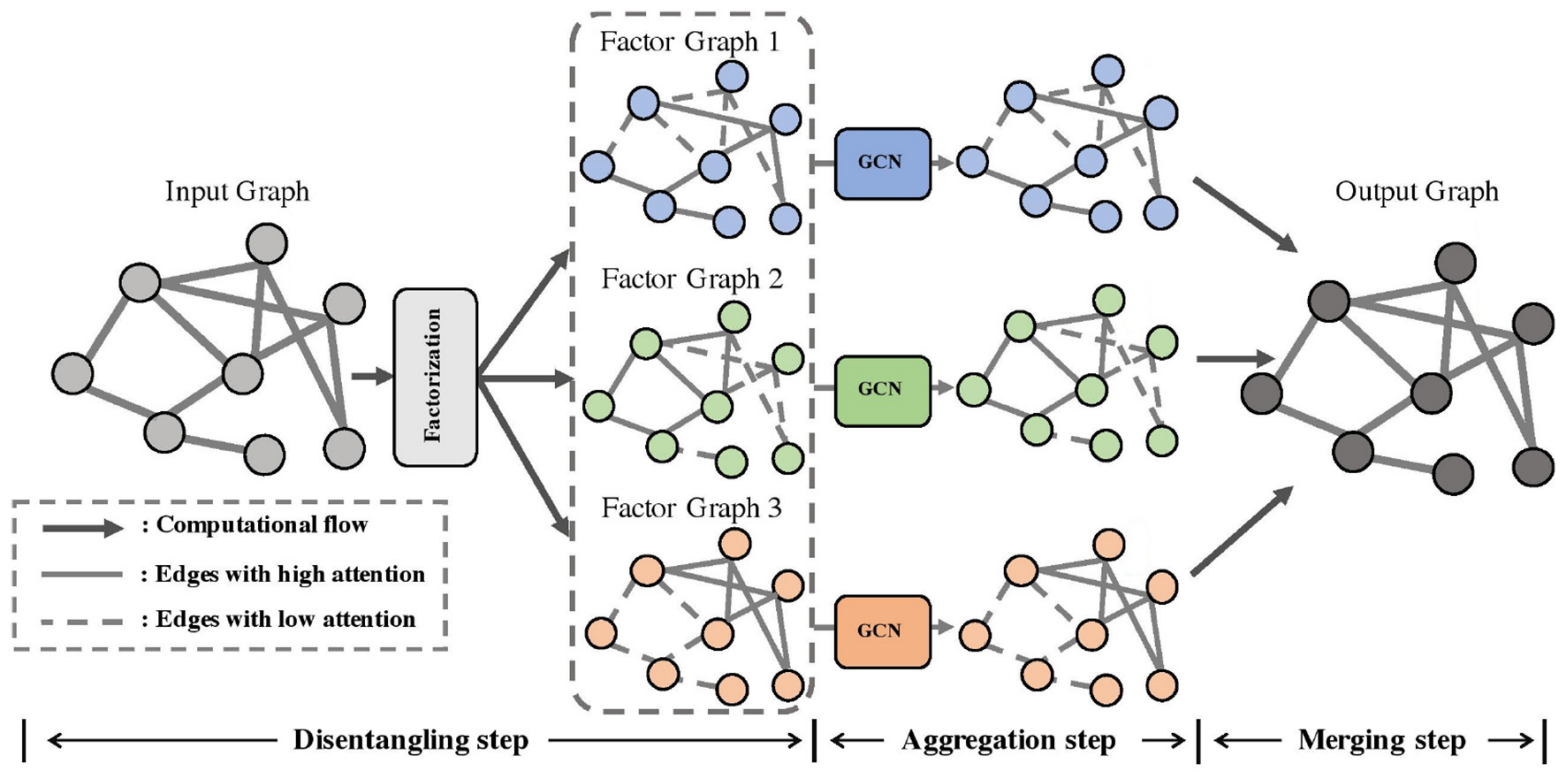
Graph convolutional network module architecture diagram.

In the PsyGraph-SSL model, the Graph Convolutional Network (GCN) module is a core component, responsible for processing social relationships and sentiment transmission among student groups, and extracting potential psychological health relationships between students through the graph structure. Figure 2 illustrates the architecture of this module. The following details its working principles and data flow.

First, the input data includes social network data and emotional fluctuation data from students. This data forms a graph structure, where each node represents a student, and the edges between nodes represent the social relationships or emotional interactions between students. The main task of the graph convolutional network module is to fuse the features of a student with the features of its neighbors through graph convolution operations to capture the mutual influence of emotions or mental health states between students. In the graph convolutional network, the node features $h_{v}^{\left(l\right)}$ are updated through multiple layers of convolution, and the update formula is:


$$\begin{array}{l} h_{v}^{\left(l+1\right)}=\sigma \left(\underset{u\in N\left(v\right)}{\sum }\frac{1}{c_{uv}}W^{\left(l\right)}h_{u}^{\left(l\right)}+b^{\left(l\right)}\right) \end{array}$$ (1)

where $h_{v}^{\left(l+1\right)}$ represents the feature representation of node *v* at the *l*+1th layer, N(v) is the set of neighboring nodes of node *v*, *W*^(*l*)^ is the weight matrix at the *l*th layer, *c*_*uv*_ is the normalization coefficient, σ is the activation function, and *b*^(*l*)^ is the bias term. Through graph convolution, the model effectively captures the dissemination of mental health information and emotional interactions among students, providing key features for subsequent mental health prediction.

After the graph convolution, the features of the student nodes are passed to the self-supervised learning (SSL) module. The task of the SSL module is to learn features from unlabeled data in order to discover potential mental health patterns. A common self-supervised learning method is contrastive learning, which aims to learn discriminative features by maximizing the similarity between similar samples and minimizing the similarity between dissimilar samples. In this paper, we assume that the features of student *i* are represented as **z**_*i*_, and the loss function of self-supervised learning is:


$$\begin{array}{l} L_{SSL}=-\underset{i,j}{\sum }\log\frac{\exp\left({\text{z}}_{i}\cdot {\text{z}}_{j}/\tau \right)}{\sum_{k=1}^{N}\exp\left({\text{z}}_{i}\cdot {\text{z}}_{k}/\tau \right)} \end{array}$$ (2)

where **z**_*i*_ and **z**_*j*_ represent the feature vectors of student *i* and student *j*, respectively, τ is the temperature parameter, and *N* is the total number of samples. Through this comparative learning objective, the model can learn important features related to students' mental health from unlabeled data, further improving its ability to predict mental health risks.

After feature update by the self-supervised learning module, the feature data is passed to the time series modeling module. The time series modeling module models the changes in students' mental health status, captures their long-term dependencies, and predicts future risks. Assuming that at time step *t*, student *i*'s mental health status is *s*_*t*_, this module uses the time series modeling function *f* to update the status, as follows:


$$\begin{array}{l} s_{t}=f\left(s_{t-1},{\text{z}}_{t}\right) \end{array}$$ (3)

where *s*_*t*_ represents the student's mental health status at time step *t*, *f* is a time series modeling function, *s*_*t*−1_ is the state at the previous time step, and **z**_*t*_ is the feature representation of the current time step. Through time series modeling, the model can predict future trends in a student's mental health status based on their historical mental health data.

Finally, the results of time series modeling are passed to the output layer, where the model generates a mental health risk score based on the student's historical emotional, behavioral, and social interaction data. Assuming the output risk score is *r*, the model converts the time series modeling output *s*_*T*_ into the final risk score through a fully connected layer. The formula is:


$$\begin{array}{l} r=W_{\text{out}}s_{T}+b_{\text{out}} \end{array}$$ (4)

where *s*_*T*_ is the mental health status at the final moment, *W*_out_ and *b*_out_ are the weight and bias of the output layer, respectively, and *r* is the final mental health risk score.

By leveraging relational modeling through graph convolutional networks, feature learning through self-supervised learning, and dynamic prediction through time series modeling, the PsyGraph-SSL model accurately assesses students' mental health status from multiple dimensions and outputs personalized mental health risk predictions. This process not only enhances the model's predictive capabilities but also provides timely mental health warnings and intervention recommendations, providing strong technical support for students' mental health management.

### 3.3 Self-supervised learning module

The Self-Supervised Learning (SSL) module is a core component of the PsyGraph-SSL model, primarily responsible for feature learning from unlabeled data. In student mental health analysis, especially when labeled data is scarce, SSL provides an effective learning approach, enabling the model to uncover potential mental health patterns from large amounts of unlabeled sentiment data. The SSL module's task is to use self-supervision to enable the model to automatically generate effective feature representations based on the inherent structure and relationships of the input data, even without the support of labeled data.

**Figure 3:**
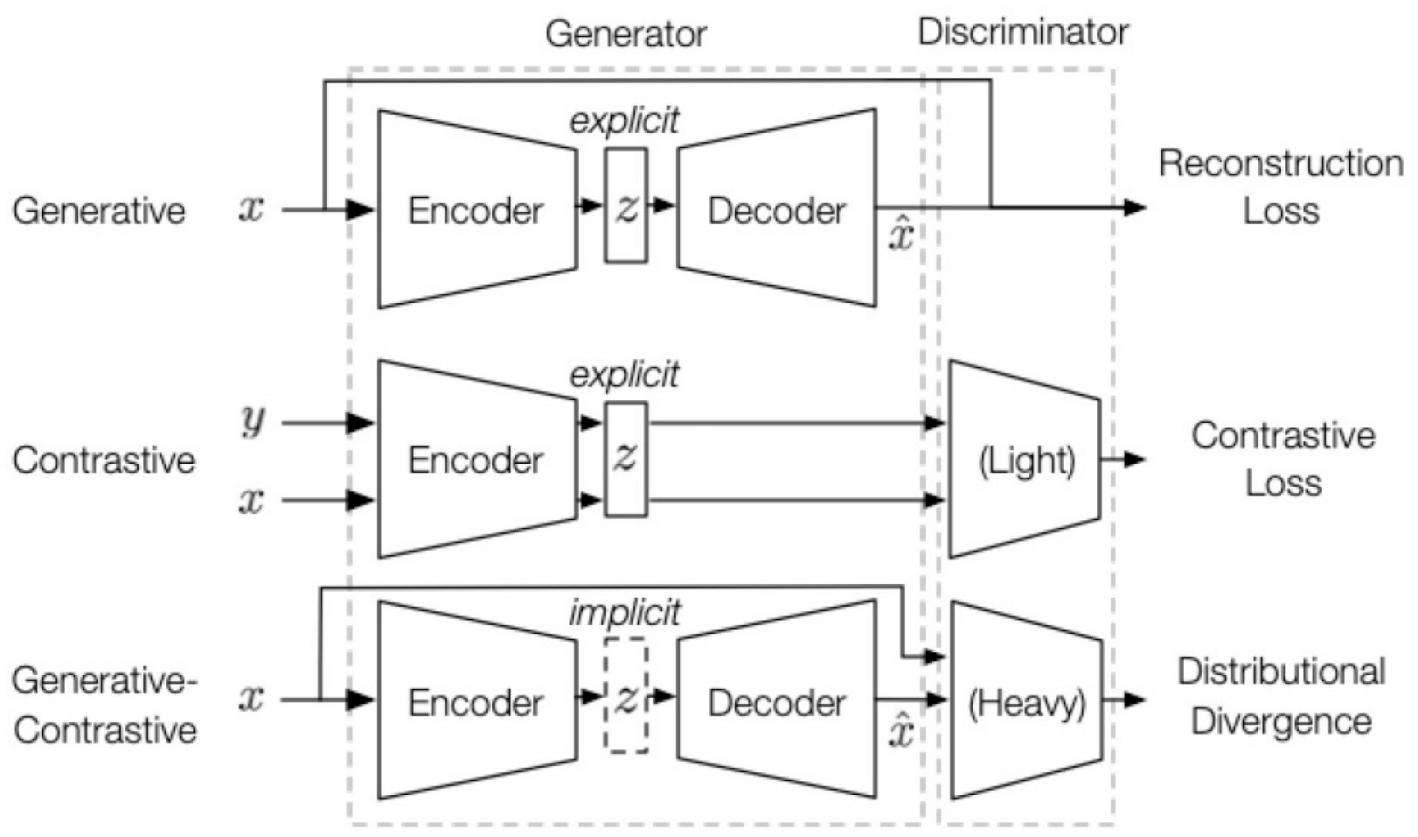
Self-supervised learning (SSL) module architecture diagram.

As shown in the Figure 3, the SSL module's workflow receives student social network data and sentiment features from the Graph Convolutional Network (GCN) module. After preliminary graph convolution operations, these data form initial feature representations of the students. These initial feature representations are then passed to the SSL module for further learning and optimization, ultimately generating deeper feature representations that provide the foundation for subsequent time series modeling and risk prediction.

In the self-supervised learning process, we adopt a contrastive learning strategy. The goal of contrastive learning is to reduce the distance between similar samples and increase the distance between different samples, so that the model can learn discriminative feature representations. In this paper, assuming that the feature representation of student *i* is **z**_*i*_, the loss function of self-supervised learning can be expressed as:


$$\begin{array}{l} L_{SSL}=-\underset{i,j}{\sum }\log\frac{\exp\left({\text{z}}_{i}\cdot {\text{z}}_{j}/\tau \right)}{\overset{N}{\underset{k=1}{\sum }}\exp\left({\text{z}}_{i}\cdot {\text{z}}_{k}/\tau \right)} \end{array}$$ (5)

where **z**_*i*_ and **z**_*j*_ represent the feature vectors of student *i* and student *j*, respectively, τ is the temperature parameter, and *N* is the total number of samples. This loss function uses contrastive learning to bring similar mental health characteristics closer together, while dissimilar characteristics are pulled apart. By optimizing this loss function, the model can learn the underlying characteristics of students' mental health. These characteristics not only improve the ability to predict mental health risks but also provide a basis for personalized intervention.

In addition, to further enhance the model's generalization capabilities, we implemented data augmentation strategies. Data augmentation techniques increase the model's adaptability to different emotional expressions by perturbing, cropping, rotating, and performing other operations on input features. In the SSL module, a common method of data augmentation is random masking and feature perturbation. The specific perturbation operations can be expressed as follows:


$$\begin{array}{l} {\hat{\text{z}}}_{i}={\text{z}}_{i}+\epsilon_{i} \end{array}$$ (6)

where ${\hat{\text{z}}}_{i}$ represents the perturbed feature representation, and ϵ_*i*_ is a perturbation term whose magnitude and direction are determined by random noise. This allows the model to learn more robust feature representations under diverse data augmentation.

In the self-supervised learning process, we also introduce a strategy that combines contrastive loss with reconstruction loss. Suppose we obtain the feature representations **z**_*t*_ of students at different time steps from historical data. We then use a model such as a temporal convolutional network (TCN) to model this time series and reconstruct their mental health status. The reconstruction loss can be expressed as:


$$\begin{array}{l} L_{recon}=\underset{t}{\sum }||{\text{z}}_{t}-{\hat{\text{z}}}_{t}|{|}^{2} \end{array}$$ (7)

where **z**_*t*_ and ${\hat{\text{z}}}_{t}$ represent the original and reconstructed feature representations, respectively, and ||·||^2^ represents the Euclidean distance. By combining contrastive learning loss with reconstruction loss, the model not only learns the inherent structure of students' mental health characteristics but also improves its ability to predict dynamic changes in mental health.

Through this series of operations, the SSL module fully utilizes unlabeled data for deep feature learning, thereby improving the accuracy and robustness of mental health risk assessment. The introduction of self-supervised learning significantly compensates for the lack of labeled data, making the PsyGraph-SSL model more adaptable and effective in practical applications.

### 3.4 Cross-modal data fusion and time series modeling

In the PsyGraph-SSL model, the cross-modal data fusion and time series modeling module primarily aims to construct a comprehensive picture of students' mental health by combining data from different modalities, such as social behavior data, affective computing data, and physiological signal data. Time series modeling is then used to capture the dynamic changes in students' mental health status. The core of this module lies in efficiently integrating these diverse data types and capturing the long-term dependencies of students' mental health status through time series modeling, thereby achieving more accurate mental health risk prediction.

First, cross-modal data fusion requires the model to address the heterogeneity of multi-source data. Data from different modalities, such as facial expression data for emotion recognition, emotional intonation in voice data, and activity trajectories in behavioral data, all provide distinct perspectives on students' mental health. To fully exploit this information, the PsyGraph-SSL model uses a feature-level fusion strategy to fuse data from different modalities within the feature space. Assuming that the data features from different modalities are **z**_1_, **z**_2_, …, **z**_*M*_, the fused features **z**_*fusion*_ can be expressed as:


$$\begin{array}{l} {\text{z}}_{fusion}=\overset{M}{\underset{i=1}{\sum }}W_{i}\cdot {\text{z}}_{i}+b \end{array}$$ (8)

where **z**_*i*_ represents the data features of the *i*th modal, *W*_*i*_ and *b* are the learned weights and bias terms, respectively, and **z**_*fusion*_ is the fused feature representation. By weightedly fusing data from different modalities, the model can integrate multiple aspects of information, thereby improving its understanding and prediction of students' mental health status.

In addition, for modeling time series data, the PsyGraph-SSL model utilizes a time series modeling module, the primary purpose of which is to capture the long-term dependencies and dynamic trends of students' mental health status. Students' emotional fluctuations and mental health status often change over time, especially during periods of long-term academic pressure or life changes. Therefore, how to effectively capture these dynamic changes becomes a key issue. In this process, the model uses time series modeling methods to model the mental health status. Assume that the student's mental health status at time step *t* is *s*_*t*_. This state can be updated by the state of the previous moment *s*_*t*−1_ and the current input feature **z**_*t*_. The update formula is as follows:


$$\begin{array}{l} s_{t}=f\left(s_{t-1},{\text{z}}_{t}\right) \end{array}$$ (9)

Here, *s*_*t*_ represents the student's mental health status at time step *t*, *f* is a time series modeling function, *s*_*t*−1_ is the state at the previous time step, and **z**_*t*_ is the feature representation at the current time step. Through this time series modeling, the model can effectively capture the temporal evolution of students' mental health status and predict the mental health risks they may face in the future.

To further improve the effectiveness of time series modeling, the model adopts a multi-head attention mechanism, which makes it more efficient in capturing long-term dependencies. In mental health risk prediction, students' emotional changes may be influenced by multiple factors, such as academic performance, social interactions, and family relationships. The influence of these factors is often intertwined and occurs at different time scales. The multi-head attention mechanism allows the model to simultaneously focus on the influence of these different factors, thereby identifying valuable patterns in complex emotional fluctuations and changes in mental health status.

The combination of time series modeling and cross-modal data fusion enables the PsyGraph-SSL model to progressively update students' mental health status at different time steps based on multi-modal data inputs and output accurate mental health risk predictions. This not only improves the timeliness and accuracy of predictions, but also provides strong data support and decision-making basis for personalized interventions. The model can provide real-time mental health assessments for each student and offer customized intervention plans based on their historical data and future predictions.

In summary, cross-modal data fusion and time series modeling modules play a crucial role in the PsyGraph-SSL model. By rationally integrating data from multiple modalities and using time series modeling to capture the dynamic changes in mental health status, the model can provide accurate, real-time predictions and intervention recommendations for student mental health management.

### 3.5 Emotion computing module

The Emotion Computing Module is designed to capture students' emotional states by analyzing multimodal data, including physiological signals, facial expressions, and speech. Specifically, the module processes voice data to identify emotional fluctuations, such as stress, anxiety, or relaxation, by analyzing speech patterns. The facial expression data is processed by Graph Convolutional Networks (GCNs) to recognize and analyze emotional cues from facial movements. These multimodal data are integrated to provide a more accurate and dynamic representation of students emotional states.

The rationale for using GCNs in facial expression recognition and voice analysis is that multimodal data allows the model to better understand the emotional fluctuations in students, which can be more effective than single-modality approaches. GCNs are particularly suitable for analyzing graph-structured data and have been shown to effectively capture relationships between different modalities, improving the accuracy of emotion detection.

To evaluate the reliability of the Emotion Computing Module, we used cross-validation techniques, comparing the models predictions to ground truth emotional state labels. The performance of the module was evaluated using accuracy, F1-score, and AUC-ROC, ensuring its robustness and reliability in predicting emotional states from multimodal data.

This approach enables the model to track real-time emotional changes in students and make dynamic mental health risk predictions, providing personalized and timely interventions based on emotional data.

## 4 Experiment

### 4.1 Datasets

In this study, we selected two public datasets—the WESAD Dataset (Schmidt et al., 2018) and the Student Wellbeing Dataset—as the foundation for our model experiments. These datasets were chosen primarily for their multimodal data characteristics and content related to student mental health, enabling them to provide multidimensional information support for mental health risk prediction. The WESAD dataset includes physiological signals, emotion labels, voice, and facial expressions, while the Student Wellbeing dataset provides emotional states, behavioral data, and academic performance. These datasets offer a comprehensive representation of students' mental health, including emotional fluctuations, behavioral patterns, and social interactions.

The data split protocol for both datasets follows a subject-held-out approach, ensuring that data from the same subject is not included in both training and testing sets, thereby preventing data leakage. This split protocol is crucial to avoid overfitting and to ensure the generalizability of the model to unseen individuals. The WESAD dataset consists of data from 15 subjects, while the Student Wellbeing Dataset contains over 1,000 student records, which provides a larger sample size for training and testing. Both datasets undergo preprocessing steps, including data normalization and feature extraction from raw physiological signals (for the WESAD dataset) and behavioral and academic data (for the Student Wellbeing Dataset). For the WESAD dataset, physiological signals are filtered to remove noise, and emotional fluctuations are labeled based on predefined categories such as relaxation, stress, and moderate stress. The Student Wellbeing Dataset labels emotional states like anxiety, depression, and stress based on the recorded behavioral data and academic performance.

#### 4.1.1 Handling missing values, outliers, and inconsistencies

In the process of data preprocessing, we addressed potential issues related to missing values, outliers, and inconsistencies:

1. Missing values:
  For both datasets, missing values were handled using mean imputation for numerical data (e.g., physiological signals or academic performance) and mode imputation for categorical data (e.g., emotional state labels). This approach ensures that the model could still benefit from the available data without introducing significant bias due to missing entries.
  In cases where the missing data exceeded a predefined threshold (e.g., more than 30% missing values in a feature), the feature was excluded from the model training process.
2. Outliers:
  We applied z-score normalization to detect outliers, particularly in the physiological signals from the WESAD dataset. Features with values beyond 3 standard deviations from the mean were considered outliers and were removed or clipped to a threshold to prevent their impact on model performance.
  For behavioral and academic data in the Student Wellbeing Dataset, IQR (Interquartile Range) methods were used to identify and handle extreme outliers, ensuring that the data remained within reasonable bounds.
3. Inconsistencies:
  Data inconsistencies, such as discrepancies between emotional state labels and physiological signals, were addressed by carefully reviewing the dataset documentation and correcting any mismatched labels.
  Any data points identified as inconsistent or incorrectly labeled during preprocessing were removed to ensure the integrity of the data used for model training.

#### 4.1.2 Data imbalance

Regarding data imbalance, while both datasets provide valuable insights into students' mental health, we observed potential class imbalances in certain categories. For example, in the WESAD dataset, the number of samples in the “relaxation” category is significantly larger than those in the “stress” and “moderate stress” categories. Similarly, the Student Wellbeing dataset may exhibit an overrepresentation of students in “normal” or “mildly stressed” categories, with fewer instances of severe mental health conditions such as anxiety or depression. To address these imbalances, we have applied class weighting in the loss function during model training, ensuring that minority classes receive more emphasis during learning. Additionally, sampling techniques such as oversampling of underrepresented classes were explored to mitigate the effects of data imbalance on model performance.

The integration of these two datasets, along with careful preprocessing and data split protocols, allows the PsyGraph-SSL model to leverage multimodal data for more accurate and timely student mental health risk assessment. This hybrid approach enhances the model's capability to predict potential mental health issues in students and supports the development of personalized interventions. Furthermore, the selection of these datasets directly influences the choice of methods—graph convolutional networks (GCN) and self-supervised learning (SSL)—which are well-suited to handle the complex, multimodal, and often sparse data, and are particularly effective for predicting dynamic and personalized mental health risks. The temporal modeling via time-series analysis is particularly important for the WESAD dataset, where mood and emotional states fluctuate over time. For the Student Wellbeing dataset, multimodal fusion techniques were essential for integrating various data sources, such as emotional fluctuations, behavioral data, and academic performance, to produce more accurate predictions.

Details of the datasets are presented in Table 1.

**Table 1:** Comparison of data characteristics between the WESAD Dataset and the Student Wellbeing Dataset.

**Dataset**	**WESAD Dataset**	**Student Wellbeing Dataset**
Data type	Physiological signals, emotion labels, voice, facial expressions	Emotional states, behavioral data, academic performance
Main content	Monitoring stress and emotional fluctuations via wearable devices	Student mental health, relationship between emotional fluctuations and academic performance
Data source	Published by a German university research team	Data collected by educational research institutions
Application domain	Emotion analysis, stress detection, mental health risk assessment	Student mental health analysis, relationship between emotional fluctuations and academic performance
Multimodal data	Yes (physiological signals, facial expressions, voice, etc.)	Yes (emotion, behavior, academic performance, etc.)
Label type	Precise emotion state labels (relaxation, stress, etc.)	Emotional fluctuations and mental health state labels
Sample size	15 subjects	1,000+ student records
Emotion classification	Relaxation, stress, mild and moderate stress levels	Anxiety, depression, stress, and other emotional states

### 4.2 Experimental results and analysis

#### 4.2.1 Model performance on the WESAD dataset and student wellbeing dataset

In this experiment, we evaluated the PsyGraph-SSL model on the WESAD and Student Well-Being datasets. To comprehensively analyze model performance, we selected several common evaluation metrics, including accuracy, precision, recall, F1 score, AUC (Area Under Curve), mean squared error (MAE), and root mean squared error (RMSE). These metrics provide different perspectives on the model's performance in classification and regression tasks. To systematically compare PsyGraph-SSL with other baseline models (such as BERT, SVM, regression models, ViT, and random forest), we calculated these metrics on both datasets. The following Table 2 summarizes the results of PsyGraph-SSL and other models on the WESAD and Student Well-Being datasets.

**Table 2:** Performance comparison on WESAD and Student Wellbeing datasets.

**Model**	**Dataset**	**Accuracy**	**Precision**	**Recall**	**F1-score**	**AUC**	**MAE**	**RMSE**
PsyGraph-SSL	WESAD	0.92	0.89	0.91	0.90	0.94	0.025	0.035
PsyGraph-SSL	Student Wellbeing	0.89	0.85	0.88	0.86	0.91	0.030	0.045
BERT	WESAD	0.87	0.84	0.86	0.85	0.90	0.050	0.065
BERT	Student Wellbeing	0.81	0.78	0.80	0.79	0.86	0.060	0.080
SVM	WESAD	0.79	0.75	0.78	0.76	0.85	0.060	0.075
SVM	Student Wellbeing	0.81	0.78	0.80	0.79	0.86	0.060	0.080
Regression	WESAD	0.80	0.77	0.79	0.78	0.87	0.055	0.070
Regression	Student Wellbeing	0.83	0.80	0.82	0.81	0.87	0.055	0.070
ViT	WESAD	0.90	0.87	0.89	0.88	0.93	0.035	0.050
ViT	Student Wellbeing	0.85	0.82	0.84	0.83	0.90	0.045	0.060
Random forest	WESAD	0.83	0.80	0.82	0.81	0.88	0.045	0.060
Random forest	Student Wellbeing	0.84	0.81	0.83	0.82	0.89	0.050	0.065

The hyperparameter settings for each model are as follows: For PsyGraph-SSL (weighted fusion method), the learning rate is 0.0001, with a batch size of 32, 50 epochs, the Adam optimizer, and a dropout rate of 0.5. For Random Forest, the hyperparameters include 100 estimators, a maximum depth of 10, a minimum samples split of 2, a minimum samples leaf of 1, and a random state of 42. ViT (Vision Transformer) uses a learning rate of 0.0001, a batch size of 16, 30 epochs, the AdamW optimizer, a patch size of 16, 12 transformer layers, a hidden size of 768, and 12 attention heads. For Regression, the settings include an SGD optimizer, a learning rate of 0.01, 100 epochs, and a batch size of 32. The SVM (Support Vector Machine) model uses an RBF kernel, C = 1.0, gamma = scale, class weights set to balanced, and a decision function shape of ovo. Finally, BERT has a learning rate of 2e-5, a batch size of 16, 3 epochs, the AdamW optimizer, a maximum sequence length of 128, and 500 warmup steps. These settings ensure consistent training conditions for each model, allowing for fair comparisons across all models.

**Figure 4:**
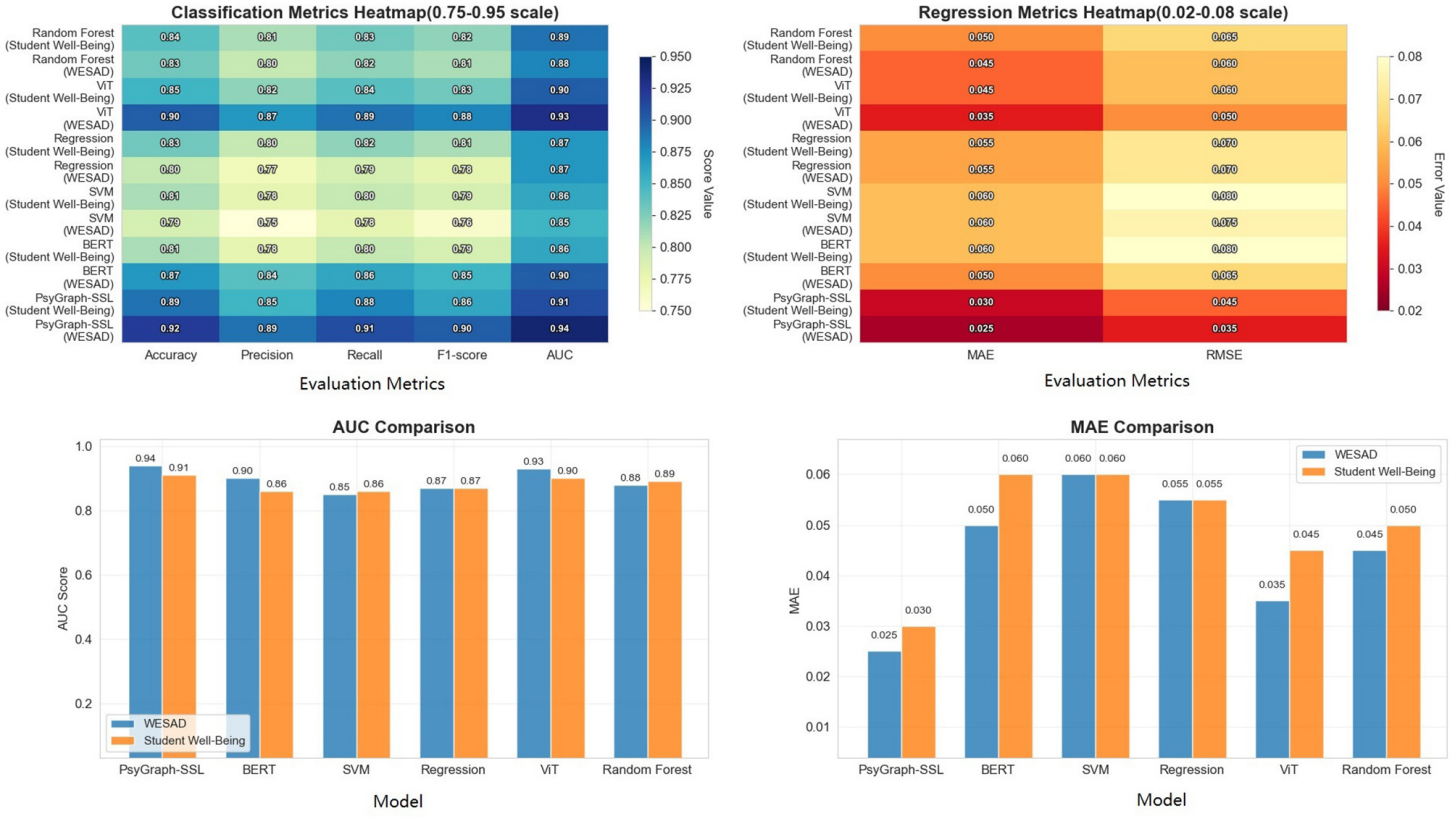
Performance comparison of PsyGraph-SSL and baseline models on WESAD and Student Wellbeing Datasets across multiple evaluation metrics.

To more intuitively demonstrate the performance differences between different models on the WESAD dataset and the Student Wellbeing Dataset, we converted the table data into a bar chart, as shown in Figure 4. The chart shows the performance of each model on various evaluation metrics. As can be seen, PsyGraph-SSL outperforms other baseline models in multiple metrics, particularly key indicators such as AUC and accuracy.

The results in Table 2 and Figure 4 provide a detailed comparison of PsyGraph-SSL with various baseline models across two different datasets: WESAD and Student Wellbeing. Overall, PsyGraph-SSL outperforms other models on both datasets in terms of accuracy, precision, recall, and F1-score, with particularly strong performance on the WESAD dataset (accuracy: 0.92, F1-score: 0.90) compared to the Student Wellbeing dataset (accuracy: 0.89, F1-score: 0.86).

Among the baseline models, BERT, ViT, and Random Forest demonstrate competitive performance, with ViT achieving the highest accuracy for WESAD (0.90) and BERT performing well on the Student Wellbeing dataset (accuracy: 0.81). However, PsyGraph-SSL consistently outperforms these models, particularly in F1-score and AUC, which are crucial for assessing the model's ability to make balanced and reliable predictions.

Notably, the SVM and Regression models exhibit lower accuracy compared to PsyGraph-SSL across both datasets, highlighting the advantages of more complex models like PsyGraph-SSL in capturing the intricate relationships between the multimodal data inputs. This comparison not only underscores the effectiveness of PsyGraph-SSL in predicting students' mental health risks but also validates its ability to integrate multiple data modalities, leading to better performance than single-modal approaches used by some baseline models.

#### 4.2.2 Model performance under different data modalities

In this experiment, we aim to evaluate the performance of the PsyGraph-SSL model under different data modalities and compare the effectiveness of feature-level weighted fusion against other common fusion methods. Specifically, we focus on analyzing the effect of integrating different types of data on model performance. While it is expected that cross-modal data fusion improves performance compared to unimodal data, the primary goal of this experiment is to demonstrate that our feature-level weighted fusion strategy outperforms other fusion approaches.

We used the following data MODALITIES:

1. Behavioral data only: Training was performed using students' social behavior data, ignoring emotional and physiological signal data.
2. Emotional data only: Training was performed using students' affective computing data (such as facial expressions and voice), ignoring behavioral and physiological signal data.
3. Cross-modal data fusion using weighted fusion: Training was performed using students' behavioral, emotional, and physiological signal data simultaneously, integrating data from all modalities using our weighted fusion strategy.
4. Cross-modal data fusion using simple averaging: This method integrates data from all modalities but uses simple averaging for fusion without weight adjustment.
5. Cross-modal data fusion using feature concatenation: In this approach, features from each modality are concatenated into a single feature vector, which is then used for training.
6. Cross-modal data fusion using neural network-based fusion: This method uses a deep learning model to learn the fusion weights for each modality during training.

The Table 3 below compares the performance of PsyGraph-SSL under different data modalities. In addition to traditional metrics such as accuracy, F1 score, and AUC, we also incorporated new evaluation metrics such as Cohen's Kappa, mean absolute percentage error (MAPE), and relative error (RE) to measure the model's performance in classification and regression tasks.

**Table 3:** Performance comparison of different data fusion methods on WESAD Dataset.

**Fusion method**	**Accuracy**	**F1-score**	**AUC**	**Kappa**	**MAPE**	**RE**
Behavioral only	0.78	0.75	0.80	0.58	0.12	0.15
Affective only	0.83	0.80	0.85	0.68	0.10	0.12
Weighted fusion	0.92	0.90	0.94	0.83	0.06	0.08
Simple averaging fusion	0.89	0.87	0.91	0.77	0.08	0.10
Feature concatenation fusion	0.86	0.84	0.88	0.72	0.10	0.12
Neural network fusion	0.91	0.89	0.93	0.80	0.07	0.09

**Figure 5:**
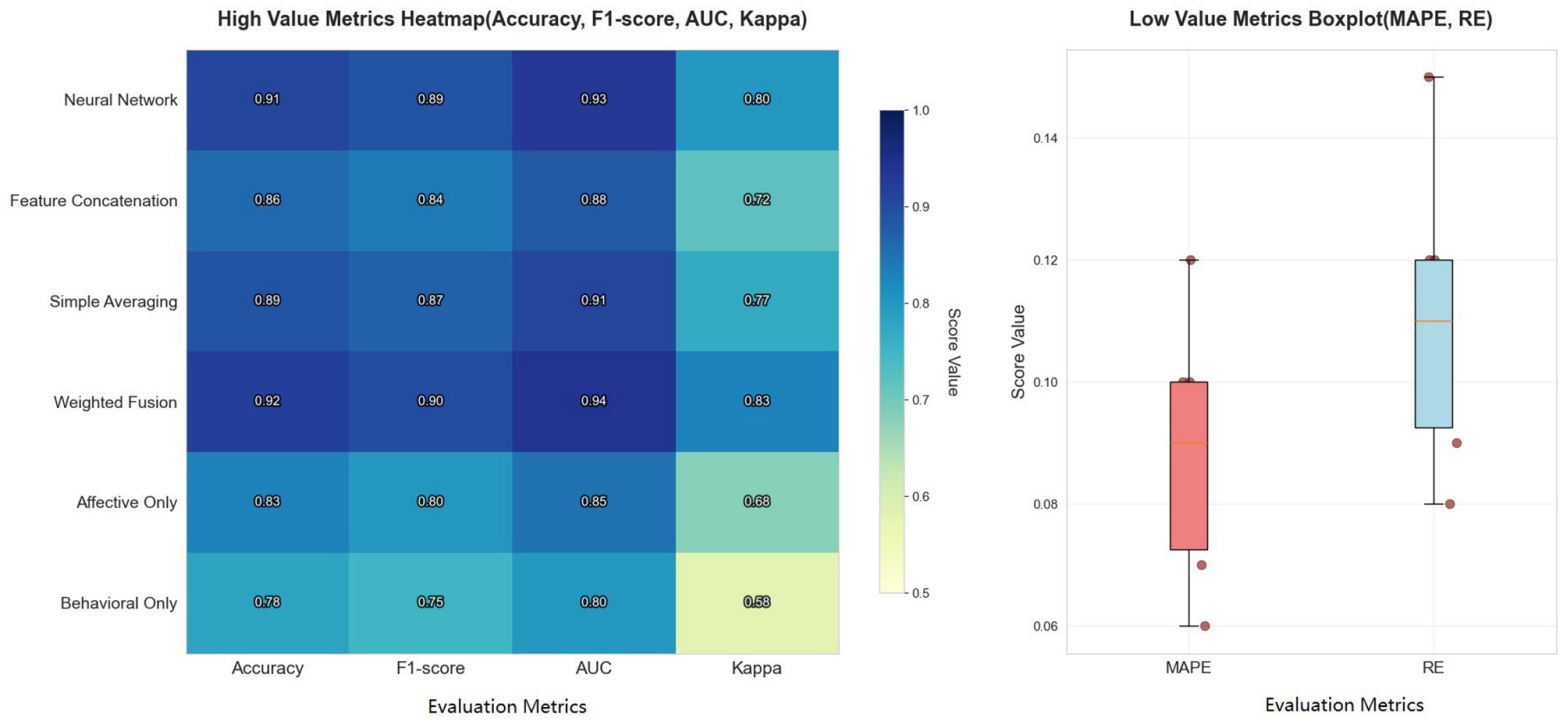
Performance comparison of different data fusion methods with various evaluation metrics.

To visually demonstrate the effectiveness of our feature-level weighted fusion, we use heatmaps to illustrate the performance changes across different fusion methods. Figure 5 shows the differences in accuracy, F1 score, AUC, Kappa coefficient, MAPE, and relative error for each fusion method, further validating the advantages of our weighted fusion strategy.

As shown in Table 3 and Figure 5, feature-level weighted fusion significantly improves model performance compared to other methods, particularly when considering high-value metrics such as accuracy, F1-score, AUC, and Kappa. Specifically, PsyGraph-SSL achieves an accuracy of 0.92 with weighted fusion, outperforming simple averaging fusion (0.89), feature concatenation fusion (0.86), and neural network fusion (0.91). This demonstrates that our weighted fusion approach, which adjusts the contribution of each modality, provides a more robust and accurate prediction of students' mental health risks.

The comparison with alternative fusion methods underscores the advantages of our weighted fusion approach, making a strong case for its use in real-world applications of student mental health assessment and intervention. The heatmap on the left of Figure 5 further reinforces this, showing that the weighted fusion method achieves the highest scores across all metrics, providing more accurate and consistent results compared to other methods.

And the boxplot on the right side of Figure 5 illustrates the performance of the fusion methods in terms of low-value metrics such as MAPE and RE, where weighted fusion shows a substantial reduction in both MAPE and RE, highlighting the stability and reliability of the models predictions. This result validates that feature-level weighted fusion is an effective approach for integrating multimodal data and improves the models ability to capture students' mental health status.

#### 4.2.3 Ablation experiments

In this experiment, we used ablation studies to verify the contribution of each module in the PsyGraph-SSL model. The core idea of an ablation study is to gradually remove or replace key modules in the model (such as the convolutional network, time series modeling, and self-supervised learning) and observe the changes in model performance to assess the importance of each module in the final performance. Ablation studies typically compare the results of the complete model with those of the model with the removed modules to explore the impact of each module on overall performance.

The Table 4 shows that each module plays a key role in the PsyGraph-SSL model. Removing the graph convolutional network (GCN) module significantly degraded the model's performance, particularly in accuracy, F1 score, and AUC, while the MAE and RMSE increased. This demonstrates the GCN module's crucial role in capturing the emotional and social relationships between students. Removing the temporal modeling module slightly decreased the model's accuracy and AUC, with relatively small changes in MAE and RMSE. However, this still demonstrates the module's importance in capturing the dynamic characteristics of students' mental health over time. Removing the self-supervised learning module significantly degraded the model's performance, particularly in accuracy and AUC, while MAE and RMSE increased. This demonstrates the importance of self-supervised learning in feature learning and optimization for unlabeled data.

**Table 4:** Ablation study results of PsyGraph-SSL components on Student Wellbeing Dataset.

**Model configuration**	**Accuracy**	**F1-score**	**AUC**	**MAE**	**RMSE**
PsyGraph-SSL (Full model)	0.92	0.90	0.94	0.025	0.035
w/o GCN module	0.87	0.84	0.89	0.050	0.065
w/o Temporal module	0.88	0.85	0.90	0.045	0.060
w/o SSL module	0.85	0.83	0.87	0.055	0.070
w/o All modules	0.75	0.70	0.80	0.075	0.090

Removing all modules (GCN, temporal modeling, and self-supervised learning) significantly degraded the model's performance, with accuracy dropping to 0.75, F1 score dropping to 0.70, and AUC dropping to 0.80. The MAE and RMSE also increased significantly. This further demonstrates the critical role of each module in the model. In particular, in multimodal data fusion tasks, removing any module results in a significant performance loss in the comprehensive analysis of emotional fluctuations, social behavior, and mental health.

The results show that the collaborative efforts of the graph convolutional network, time series modeling, and self-supervised learning modules significantly improve the model's accuracy and robustness. Removing any one module results in a significant performance degradation, especially in complex multimodal data fusion tasks. Each module plays an irreplaceable role in the model's successful application.

#### 4.2.4 The impact of hyperparameter tuning on model performance

In deep learning models, the choice of hyperparameters has a crucial impact on the model's training process and final performance. Different hyperparameter configurations can lead to significant differences in model training speed, convergence stability, and final prediction accuracy. Therefore, to further optimize the performance of the PsyGraph-SSL model, we tuned several key hyperparameters and evaluated their impact on the model's performance on the student mental health risk prediction task.

In this experiment, we adjusted the hyperparameters of the PsyGraph-SSL model to investigate their impact on model performance. The performance of deep learning models depends heavily on the choice of hyperparameters, and appropriate hyperparameter configurations can significantly improve model training performance and prediction accuracy. In this experiment, we focused on three key hyperparameters: learning rate, batch size, and number of graph convolution layers. By optimizing these hyperparameters, we further improved the model's performance on the student mental health risk prediction task.

The following Table 5 shows the performance of the PsyGraph-SSL model under different hyperparameter configurations. We measure the impact of different hyperparameter configurations on model performance by evaluating multiple evaluation metrics, including Matthews correlation coefficient (MCC), log loss, weighted average precision, specificity, MAE (mean absolute error), and RMSE (root mean square error).

**Table 5:** Hyperparameter tuning results with different configurations.

**Learning rate**	**Batch size**	**GCN layers**	**MCC**	**Log loss**	**Weighted precision**	**Specificity**	**MAE**	**RMSE**
0.001	32	3	0.87	0.25	0.91	0.94	0.025	0.035
0.0005	32	3	0.84	0.30	0.89	0.91	0.030	0.045
0.005	32	3	0.80	0.35	0.85	0.88	0.035	0.050
0.001	16	3	0.83	0.28	0.90	0.92	0.028	0.042
0.001	64	3	0.80	0.40	0.86	0.87	0.032	0.048
0.001	32	2	0.82	0.33	0.89	0.90	0.027	0.038
0.001	32	4	0.85	0.29	0.92	0.93	0.026	0.037

**Figure 6:**
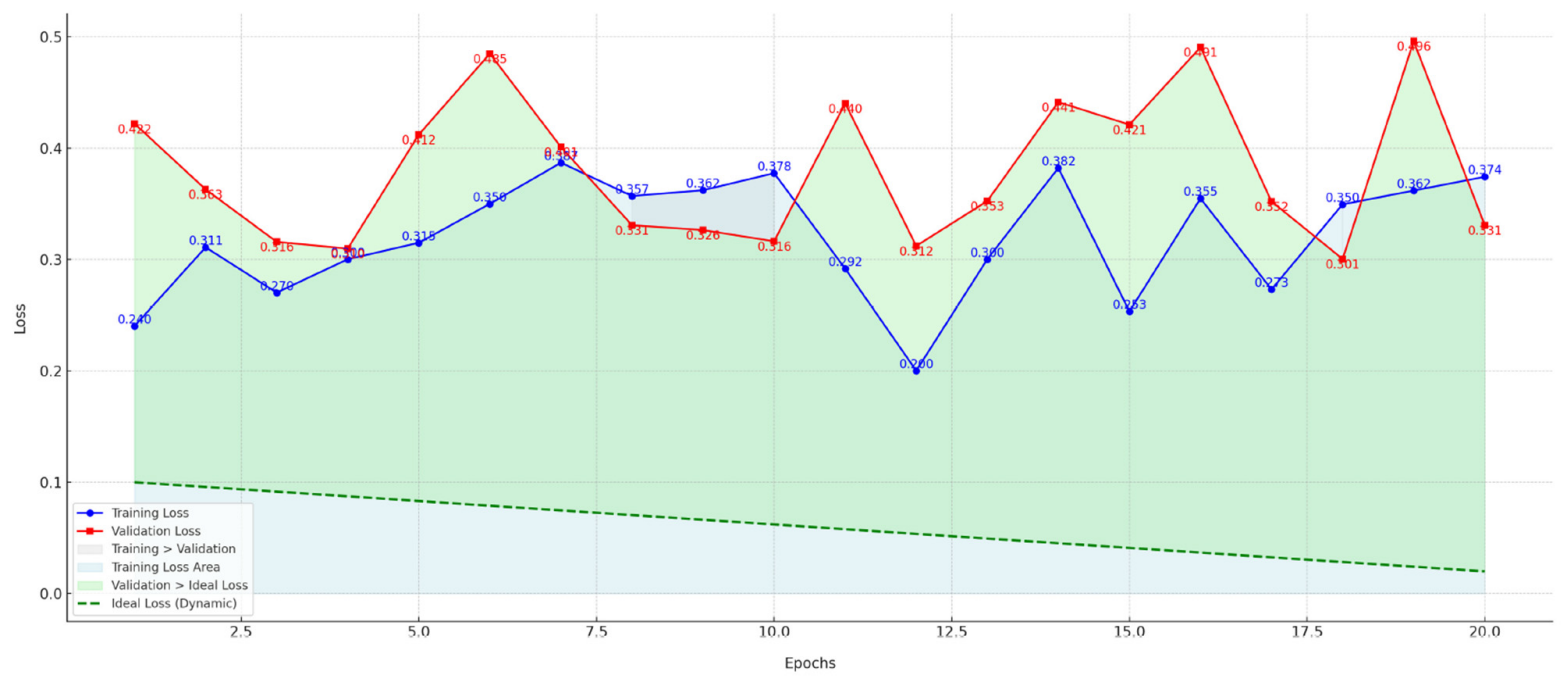
Training and validation loss curves for PsyGraph-SSL over 50 epochs with a learning rate of 0.0001 and batch size of 32.

The Figure 6 shows the training loss and validation loss curves for different hyperparameter configurations over the training epochs. This figure includes the following key points:

1. Training loss and validation loss curves: The blue and red curves represent the training loss and validation loss, respectively, and provide a visual representation of the model's convergence during training. A gradual decrease in the training loss curve indicates that the model is gradually optimizing. The trend of the validation loss curve reflects the model's performance on the validation set.
2. Ideal loss line: The green dashed line represents the ideal loss value (dynamic ideal loss), which simulates a goal that gradually decreases during training. Ideally, the model's loss should gradually approach this ideal loss value. By comparing it with the ideal loss line, we can see how the model approaches this goal during training.
3. Filled area: The filled area in the figure helps us visually visualize the gap between the model's training and validation performance. Training loss area (light blue): Shows the area of loss during training, indicating the magnitude of the decrease in training loss. Validation Loss vs. Ideal Loss (light green): Indicates the gap between validation loss and ideal loss, further highlighting the deviation between the model and the target.
4. Data annotation: The loss values (training loss and validation loss) for each training round are annotated next to the data points, making it easy to see the specific numerical changes.

Although the initial curves do not show a clear gradual decrease in Figure 6, stability begins to emerge in later epochs, particularly around epoch 30, as indicated by the slight flattening of the curves. The total number of epochs in this experiment was 50. The observed fluctuation in the earlier epochs may be due to the models adaptation to the data, which stabilizes as the model learns to generalize better over time. Additionally, these curves were generated using a learning rate of 0.0001 and a batch size of 32, as specified in the hyperparameter settings. Future experiments with different learning rates or batch sizes could provide further insights into how these hyperparameters affect the loss convergence and model performance.

By comparing the experimental results of different hyperparameter configurations, we found that the learning rate is the most critical hyperparameter. At a higher learning rate (such as 0.005), the model's performance dropped significantly, with the MCC value dropping to 0.80 and the Log Loss increasing. This indicates that excessively high learning rates can lead to unstable training and may miss the optimal solution. A moderate learning rate (0.001) resulted in a more stable loss decrease during training and achieved optimal performance across all metrics, with an MCC of 0.87, a weighted average precision of 0.91, and a specificity of 0.94, demonstrating efficient and accurate training. Adjusting the batch size also affected model performance. Smaller batch sizes (such as 16) can improve MCC and weighted average precision, but larger batch sizes (64) can slow down the model convergence and reduce performance. This indicates that smaller batches can accelerate the model training process and improve its generalization ability.

## 5 Discussion and conclusion

In this paper, we proposed PsyGraph-SSL, a novel model for student mental health risk analysis that integrates graph convolutional networks (GCN), temporal modeling, and self-supervised learning (SSL). Our motivation stemmed from the growing need for accurate and early prediction of student mental health using multiple data sources, such as behavioral, emotional, and physiological signals. Traditional methods struggle to capture the complex interdependencies among these data types and temporal dynamics. PsyGraph-SSL addresses these challenges by combining multi-modal data and learning temporal dependencies, aiming to provide a comprehensive tool for monitoring student mental health.

Our experiments demonstrated that PsyGraph-SSL outperforms baseline models on the WESAD and Student Wellbeing Dataset across multiple metrics, including accuracy, F1 score, and AUC. The model showed strong performance in predicting emotional and behavioral fluctuations, highlighting the effectiveness of multi-modal data fusion and temporal modeling. Ablation studies confirmed the contributions of GCN, temporal modeling, and self-supervised learning to the overall performance. Additionally, hyperparameter tuning experiments showed the models sensitivity to learning rate, batch size, and graph convolution layer count, contributing to its improved performance.

Despite these promising results, there are some limitations. First, while PsyGraph-SSL performed well on the datasets used, its generalization to other datasets with different types of student data remains uncertain. Further testing across diverse datasets is needed to assess its robustness and scalability. Second, the reliance on multi-modal data fusion may present challenges in real-world applications, particularly with incomplete or noisy data, which could impact performance. Addressing these issues will be crucial to enhancing the models practical utility.

Future work will focus on two main areas. First, we aim to expand the dataset by incorporating additional data sources, such as academic performance, social media activity, and real-time physiological signals. This will improve the models robustness and generalization. Second, we plan to explore advanced techniques for data imputation and denoising to better handle real-world noisy or missing data. We also aim to implement real-time monitoring systems that can provide continuous alerts and interventions based on the predictions of PsyGraph-SSL.

In conclusion, this research introduces an innovative approach to predicting and analyzing student mental health risks by leveraging multi-modal data and temporal dependencies. While the model shows strong potential for early detection and intervention, there is still room for improvement in generalization and robustness. With future enhancements, we aim to develop a more adaptable and reliable system for student mental health monitoring.

## Data Availability

The original contributions presented in the study are included in the article/supplementary material, further inquiries can be directed to the corresponding authors.
